# Principals, Agents, and the Intersection between Scientists and Policy-Makers: Reflections on the H5N1 Controversy

**DOI:** 10.3389/fpubh.2014.00109

**Published:** 2014-08-05

**Authors:** Keelie Lyn Elektra Murdock, David Koepsell

**Affiliations:** ^1^Rathenau Institute, The Hague, Netherlands; ^2^Department of Philosophy, Delft University of Technology, Delft, Netherlands

**Keywords:** principal–agent problems, H5N1, dual-use, science-policy

## Introduction

When the news broke that Ron Fouchier and his research team at Erasmus Medical Center (MC) in the Netherlands had genetically modified the highly pathogenic avian influenza (HPAI) H5N1 virus and that it had acquired the ability to transmit between mammals, it was a story of scientific discovery and progress and an exciting new development in the international effort to prevent the next pandemic. However, public anxieties and national security concerns would soon become a point of contention between virologists and biosecurity experts in the media and in highly politicized discussions about science-policy. In considering the controversy and the conflicts between scientists and policy-makers, we propose that regarding the situation as a principal–agent problem can yield useful analytical results.

Principal–agent problems occur when two parties that are driven by competing self-interest negotiate the terms of a relationship or contract and act together toward a mutually defined end but an informational asymmetry provides one party (typically the agent) with certain advantages, thus creating tensions. Principal–agent theory provides a template of relational action and the conditional effects of actions in contract situations defined by a functional differentiation, such as between scientists and the government (on behalf of its citizens). Exploring the science-policy nexus from this perspective may further efforts to develop effective policies that address dual-use concerns in the life sciences by offering insights into methods of dispute resolution and the effective design of institutional mechanisms that balance the interests of the parties involved thereby level the playing field.

## A Principal–Agent Problem?

Drawing from traditional tools of economic analysis and the theory of rational actors, principal–agent models provide a useful heuristic to explore the behavior of actors and intuitions engaged in contractual relations when there is informational asymmetry and incompletely overlapping or opposing goals. Based on the assumption that the principal requires an agent with a specific skill-set to perform specific actions or functions and both actors enter an agreement to further their interests, the relationship is based on a division of labor. The problem is one of delegation. With imperfect or incomplete information about the interests or the abilities of the applicant, it is possible that the principal will select the wrong agent to pursue its goals and endure the opportunity cost (i.e., adverse selection). If the principal and the agent enter into an agreement, the agent is offered an opportunity to gain from specialization and the informational advantage that it provides, including the conditional authority to act on behalf of the principal, the concomitant financial and professional rewards and some degree of autonomy. The principal must in turn relinquish valued resources and ensure that they receive an adequate return on the investment, in terms of productive labor and output realization.

The central difficulty for principals during the post-contract stage of the relationship, as articulated by Arrow (1), is that “by definition the agent has been selected for his specialized knowledge and the principal can never hope to completely check the agent’s performance” (2). The principal must thus bear the risk that the agent will act definitely or in ways that will have consequences for which it will be liable (i.e., moral hazard). The principal also has an incentive to minimize the risk of the transaction by reducing uncertainty and negative externalities (i.e., agency costs). This can be achieved via the strategic introduction of information revelation and generation mechanisms or by offering the agent incentives based on self-interest (such as pay by performance or profit sharing schemes) to create better alignment and ensure better communication and cooperation from the start (3, 4).

### Rules of the game

As the principal in the relationship between the scientists and the US government, the National Institutes of Health (NIH) retained the Erasmus MC Department of Virology to conduct research in support of the US Department of Health and Human Services (HHS) Pandemic Influenza Plan (5). The terms of the contract between the NIH and the Centers of Excellence for Influenza Research and Surveillance (CEIRS) are defined in the solicitation document and include the provisions for all grant recipients, including foreign institutions, to comply with NIH policies and relevant US regulations (6).

The Erasmus MC Department of Virology was selected to perform research within the CEIRS network because the research proposal defined a problem of mutual interest, in scientific terms as well as in terms that were consistent with the objectives of pandemic preparedness^1^. In addition, Ron Fouchier is specialized in the pathogenicity of respiratory viruses and has an established publication record in this particular domain of research expertise, signaling to the scientific community and funding agencies alike his competency in virology. However, the decision to conduct research on the transmissibility of avian influenza was not a decision that followed directly from NIH funding but had been under consideration at Erasmus MC since the initial detection of the virus in 1997 (7). It was thus against a backdrop of scientific uncertainty and questions about an emerging virus that that Fouchier defined his research questions and the methods of experimentation (8). Given some industry affiliations (including patents), an interest in future commercial applications can also be presumed^2^.

### Controversy

At an influenza conference, Dr. Fouchier announced that a “stupid” experiment succeeded in creating an airborne strain of the virus and the result was “very bad news” (9). The media had a field day with the story about what was in his terms, “probably one of the most dangerous viruses you can make” (10). It was soon revealed that Fouchier had submitted a paper to *Science* and intended to openly publish the intricate details. The headlines that ensued expressed strong reactions and objections to the research and the publication, including references from reputable biosecurity experts (11).

Upon review by the US National Scientific Advisory Board for Biosecurity (NSABB), the HHS was advised to request redacted versions of two manuscripts, including the paper co-authored by Ron Fouchier and a similar paper submitted to *Nature* by Yoshihiro Kawaoka from the University of Wisconsin-Madison. According to an NIH Press Statement, the board’s recommendations called for key details to be removed to prevent the replication of the experiments “by those who would seek to do harm” (12).

The Chair of the NSABB further clarified that the recommendation was based on the perception that the potential negative consequences of publishing the manuscripts outweighed the benefits. The intention, however, was neither to restrict the dissemination of information to persons with a legitimate need to know nor for the US to dominate what was essentially a global issue. Rather, the US government was also considering a mechanism to enable secured access and would pursue “broad” discussions with “global leaders” on matters of policy, science, and public health (13). This point gave credence to concerns that the limitations would interfere with scientific progress and public health preparedness, particularly the recently established and hard wrought Pandemic Influenza Preparedness (PIP) Framework of the World Health Organization (WHO).

The scientists connected to the contentious studies and the Editor-in Chief of both journals conceded to the request for a redaction but on the condition that further progress would be made on matters of policy. The former imitated a “pause” on H5N1 gain-of-function experiments to buy time for scientists to communicate with the public and policy-makers, for governments to consider policy solutions and for the scientific community to assemble and discuss the issues in an international forum (14). The latter indicated that it is next steps would rest on the US government capacity to share the omitted details (15).

At a “technical consultation” hosted by the WHO in Geneva, it was decided by consensus that the research was essential, that the papers should be published without restrictions, and that limiting access to the research results was missing a practical vision (16)^3^. The NSABB was thereafter requested on behalf of the US government to review two new manuscripts. Clarifications provided by the authors and “non-public data” discussed at the WHO meeting were named as key factors influencing the NSABB decision to revisit the matter (17).

The NSABB concluded by a majority rule that the publications should be “communicated in full” (18). The consideration of new epidemiological data and classified security information relevant to the risk-benefit calculation and the release of a new United States Government Policy for Oversight of Life Sciences Dual-Use Research of Concern (DURC) informed the discussion and influenced the decision. The moratorium, however, was upheld until the following year as NIH funded scientist awaited pending changes in the funding policy for transmissibility studies (19).

## Discussion

Within constraints of science-policy, the roles of the government and of science are institutionally mediated (20). Governments are appointed to serve the interests of the public and retain scientific information to these ends. Scientists are delegated with the authority to conduct specialized research in pursuit of particular goals and missions. The government is empowered to dictate how the agent should act and define the limits of autonomy. Scientists, however, have an informational advantage because they are on the front lines of knowledge development and have other motivations influencing their decisions. The relationship manifests as a dynamic series of moves taken by the principal and the agent to protect their respective interests, beginning with the negotiation of the contract^4^.

The contract between the NIH and Ron Fouchier aligned their interests on pandemic preparedness. However, different preferences about how the research results should be communicated combined with insufficient incentives from the government can be perceived as creating a moral hazard. Fouchier’s colorful description of his research and outspoken views on openness in science landed him in the political hot seat but the risk was not borne by him. Rather, it is in the interest of scientists to increase their visibility within the research community and to publish in prestigious journals because science communication facilitates research progress and because it can improve their career prospects.

Had the NIH thoroughly considered the objectives of the research methods and the implications in relation to existing dual-use concerns or had Fouchier been compelled to be more explicit about the potential outcomes, the controversy and at least some of the agency costs could have been mitigated.

The agency costs include the research delayed by the extension of the moratorium, the drafting of comprehensive changes in government policies and the implementation and performance thereof. The consequences of the monitoring and new bureaucratic rules, however, will also be felt by the scientists engaged in this type of research.

While not a typical adverse selection problem, the international dimension to this problem raises questions about the relationship between principals and foreign agents and whether discrepancies between national dual-use policies and legal requirements will impact future funding decisions.

The Netherlands for instance requested an export license for Fouchier’s manuscript, which delayed the publication and complicated the redaction option (21). In addition, the new US government policies introduced selection mechanisms that exclude certain research projects, including those that cannot be openly communicated or conducted in civilian (non-classified) research laboratories. The review and oversight procedures may also provide a disincentive for scientists to pursue particular research projects or seek certain funding opportunities. This may impact the international marketplace for research grants. Ron Fouchier for instance has claimed that he “would not be silenced by the Americans anymore,” and has continued his studies using funding from the European Union (22).

We propose that exploring the complex entanglement of decisions taken by the many principals and agents in this case, including those on the international level such as the WHO, can provide further insight into the tensions between scientists and policy-makers and indicate what can be done to help all parties involved to achieve their goals. The analytical framework can also help alleviate conflicts and prevent similar problems. For instance, if principals and agents have non-aligned interests, and a stand-off may prevail instead of progress. In addition, when incentives are strong, there is essentially no need for monitoring, which can be an ineffective mechanism if it excessive constraint are placed on the agent (23). These issues have worked to the detriment of the relationship between the principal and the agent in the H5N1 case and of the general public, which may have lost confidence in the institutions involved. This may well have been avoided if a more meaningful negotiation process was pursued or boundary spanning mechanisms were developed to bridge the asymmetry of information, which can improve trust and transparency and increase the possibility of interest mediation (20).

## Conflict of Interest Statement

The authors declare that the research was conducted in the absence of any commercial or financial relationships that could be construed as a potential conflict of interest.

## Footnotes

^1^The identification of mutations influencing influenza transmission can facilitate the development of medical countermeasures such diagnostics, vaccines, and drug therapies.

^2^At the time the paper was submitted, Fouchier was a part-time employee, the Chief Scientific Officer (CSO) and a shareholder in ViroClinics Biosciences, a lab that conducts virology research in support of clinical trials, diagnostics, and medical treatments. Conflicts of interest in this case, however, were avoided by letting the shares to the Stichting Administratiekantoor Erasmus Personeelsparticipaties (a financial institution established to hold shares for Erasmus University staff).

^3^According to the report, “the group recognized the difficulty of rapidly creating and regulating such a mechanism in light of the complexity of international and national legislation,” and thus decided that it was not viable.

^4^In scholarly discussions about the dynamics of science-policy, this process is referred to as “boundary-work” and serves to stabilize the boundary between science and politics.
